# Supplementary material for: Environmental memory boosts group formation of clueless individuals
Source: Nat Commun. 2023 Nov 13;14:7324. doi: 10.1038/s41467-023-43099-0 (PMC10643543; doi:10.1038/s41467-023-43099-0)
Supplement: Supplementary file 1 — Supplementary Information [file 41467_2023_43099_MOESM1_ESM.pdf]

Supplementary Information for Environmental Memory Boosts Group Formation of Clueless Individuals

Cristóvão S. Dias[1,2], Manish Trivedi[3], Giovanni Volpe[4*], Nuno A. M. Araújo[1,2*] and Giorgio Volpe[3*]

[1]Departamento de Física, Faculdade de Ciências, Universidade de Lisboa, 1749-016 Lisboa, Portugal.

[2]Centro de Física Teórica e Computacional, Faculdade de Ciências, Universidade de Lisboa, 1749-016 Lisboa, Portugal.

[3]Department of Chemistry, University College London, 20 Gordon Street, WC1H 0AJ London, United Kingdom.

[4]Department of Physics, University of Gothenburg, Origovägen 6B, SE-41296 Gothenburg, Sweden.

*Corresponding author(s). E-mail(s): giovanni.volpe@physics.gu.se; nmaraujo@fc.ul.pt; g.volpe@ucl.ac.uk;

Supplementary Figures

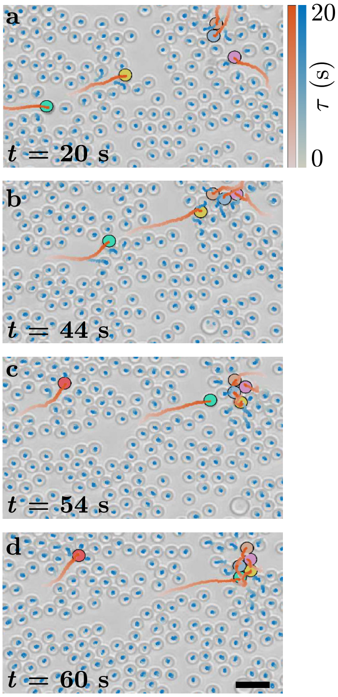

Supplementary Figure 1 Additional example of group formation of active particles via environmental memory. (**a-d**) Time sequence showing (**a-b**) a light-activated Janus particle (yellow) joining a newly formed group of three active particles (orange, purple and blue) after having burrowed through a crowded environment of SiO_2 passive particles (densities $\rho_a = 1.1\%$ and $\rho_p = 37.5\%$ as in Fig. 1). (**c-d**) A nearby Janus particle (cyan) reuses this path before joining the same cluster. In each image, 20 s-long trajectories are shown for both active (red colour scale) and passive (blue colour scale) particles; t represents the time of each frame and τ the time along each trajectory. Scale bar: 10 μm.

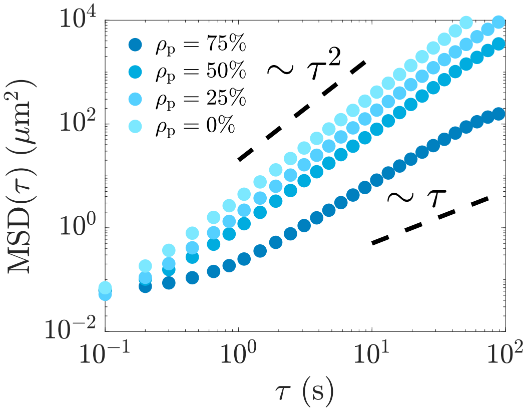

Supplementary Figure 2 Mean square displacements of Janus colloids for different $\rho_{\rm p}$. Average mean square displacements (MSDs) of active colloids ($\rho_{\rm a} = 1.1\%$ as in Fig. 1 and Supplementary Fig. 1) self-propelling through different densities $\rho_{\rm p}$ of passive colloids. The MSDs as a function of lag time τ show that the area explored by the active colloids decreases when $\rho_{\rm p}$ increases due to the additional resistance imposed by the passive colloids on the active particles' motion. The two dashed lines show persistent ($\propto \tau^2$) and diffusive ($\propto \tau$) behaviour, respectively, for reference. Each experimental MSD curve was obtained as an ensemble average over the trajectories of at least 143 Janus particles. Source data are provided as a Source Data file.

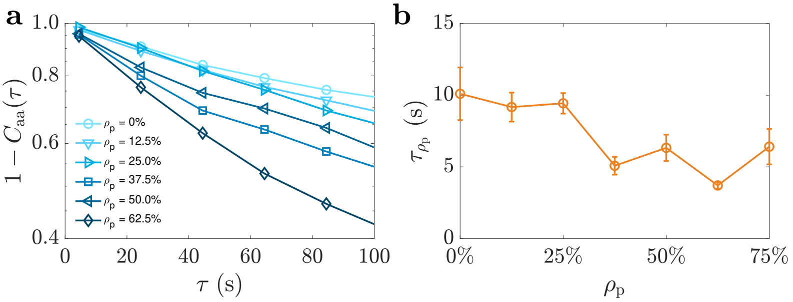

Supplementary Figure 3 Path revival function and lifetime at different densities $\rho_{\rm p}$ of passive colloids. (**a**) The path revival function $1 - C_{\rm aa}(\tau)$ calculated for a density of active particles $\rho_{\rm a} = 1.1\%$ at different densities $\rho_{\rm p}$ of passive colloids shows the probability that a region visited by an active colloid will be revisited by some other active particle within a given lag time τ before a group is formed. The faster the decay of this function, the longer a previously formed path survives in time as its reuse stabilises it for longer before it closes due to thermal fluctuations. Each experimental revival function was obtained as an ensemble average over the trajectories of three videos at $\rho_{\rm a} = 1.1\%$ per each $\rho_{\rm p}$ value. (**b**) Fitting the previous trends to an exponential $1 - C_{\rm aa}(\tau) = \exp\left(-\tau/\tau_{\rho_{\rm p}}\right)$, we can calculate an effective path revival lifetime $\tau_{\rho_{\rm p}}$ as a function of $\rho_{\rm p}$, which sees an approximately factor-two decrease after intermediate values of $\rho_{\rm p}$ starting from $\rho_{\rm p} = 37.5\%$ despite the particles' motion becoming hampered by higher levels of crowding (Supplementary Fig. 2). The error bars are an estimate of the uncertainty of the fitting. Source data are provided as a Source Data file.

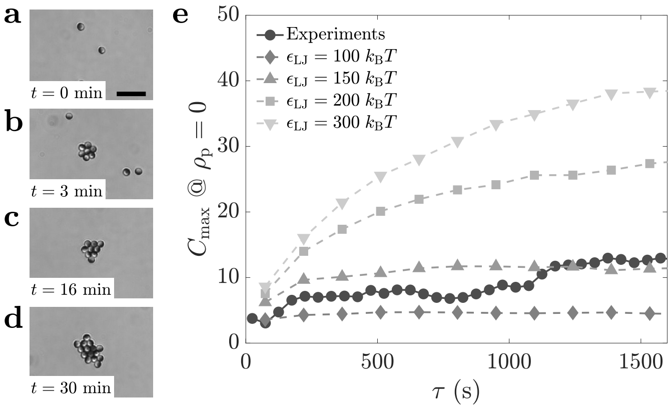

Supplementary Figure 4 Estimation of the Lennard-Jones potential's depth, ϵ_{LJ}. (**a-d**) Snapshots of a time sequence showing the formation of a group in a homogeneous environment (density of passive colloids $\rho_p = 0\%$). Scale bar: $20\,\mu$m. (**e**) The change of this group's size C_{max} in time allow us to estimate the depth ϵ_{LJ} of the Lennard-Jones potential between active colloids by matching the simulations to this experimental trend. The experimental results are better reproduced for $\epsilon_{LJ} = 150\,k_BT$. Source data are provided as a Source Data file.

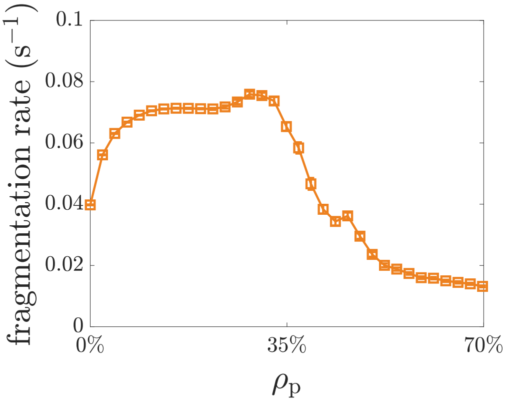

Supplementary Figure 5 Fragmentation rate as a function of ρ_p. Total fragmentation rate accounting for all possible fragmentation processes (calculated as the number of fragmentation events per unit of time) for the calculations shown in Fig. 5. This rate is approximately constant for low and intermediate values of density ρ_p of passive colloids, i.e. where the largest groups are observed in our experiments, and drops fast at higher values. This supports aggregation, rather than fragmentation, as the leading factor in the dynamics of group formation observed in Fig. 2. Error bars represent one standard error around the average values. Source data are provided as a Source Data file.
